# Multiple osteochondromas of the cervical spine, a potential cause of radiculopathy in the elderly: A case report and review of literature

**DOI:** 10.1016/j.ijscr.2020.03.018

**Published:** 2020-03-28

**Authors:** Andhika Yudistira, Yasushi Fujiwara, William Putera Sukmajaya, Ray Asaf Hexa Pandiangan, Muhammad Abduh

**Affiliations:** a Department of Orthopaedics and Traumatology, Saiful Anwar General Hospital-Universitas Brawijaya, Malang, Indonesia; b Department of Orthopaedic Surgery, Asa Citizens’ Hospital, Hiroshima City, Japan

**Keywords:** Case report, Cervical spine, Elderly, Osteochondroma, Radiculopathy

## Abstract

**Introduction:**

Osteochondroma is the most prevalent type of bone tumour, often arising in the long bones. Most are found in the third decade of life, and rarely in the axial skeleton of elderly patients. This case report aims to highlight the possibility of this differential diagnosis in an elderly patient with symptoms of radiculopathy, aside from degenerative cause.

**Presentation of case:**

A 76-years-old woman presented to the authors’ hospital with complaints of pain and numbness of the left suboccipital and preauricular region. There was hypoesthesia of left C2 and C3 dermatome without any signs of myelopathy. CT-scan and MRI showed an expansile bone lesion from the posterior arch of C1 and lamina of C2. The patient underwent laminectomy of C1 and hemilaminectomy of C2. Postoperative histopathologic examination showed the features of osteochondroma. Radiologic follow-up after nine months showed no sign of recurrence. After eighteen months, the patient was ambulatory with complaints of fingers numbness and moderate neck pain.

**Discussion:**

Osteochondroma usually arises in long bone metaphysis. This lesion normally ceases to grow with growth plate closure, but other findings suggest it may continue to grow beyond skeletal maturity. About 29.5% of all osteochondroma of the spine would cause radiculopathy, and 27% would cause myelopathy. The cervical spine osteochondroma is usually treated by *en bloc* resection through posterior approach.

**Conclusion:**

Osteochondroma of the cervical spine in the elderly is rare. However, this diagnosis could be considered as a possible cause of radiculopathy in this age group aside from other degenerative causes.

## Highlights

• Osteochondroma is the most common type of bone tumor.
• It rarely arises on the cervical spine.
• It rarely occurs in the elderly.
• Although rare, it may be considered as a differential diagnosis of radiculopathy among the elderly.

## 1 Introduction

Osteochondroma is the most prevalent benign bone tumour, characterized by an osteocartilaginous cap and bone marrow tissue continuous to the underlying bone [1]. This lesion accounts for 10% of all bone neoplasm and 35% of benign bone lesions. It is commonly found on the appendicular skeleton, but it may rarely present on axial skeleton including on the spine [2].

Spinal osteochondroma frequently occurs in the third decade of life and rarely affects the elderly. The most common predilection of this benign neoplasm is on the cervical spine, followed by lumbar, thoracic, sacrum, and coccyx; it most commonly affects the posterior column of the spine [3,4].

We present an unusual case of symptomatic multiple osteochondromas in an elderly patient arising from the posterior arch of C1 and lamina of C2. This work is reported in line with SCARE 2018 criteria [5].

## 2 Case report

A 76-year old female presented to the authors’ hospital outpatient clinic with pain and numbness of the left suboccipital and preauricular region which persisted for the last six months. There was hypoesthesia of the left C2 and C3 dermatome; there were no signs or symptoms of myelopathy (spinal cord compression). The patient’s past medical and familial history was not remarkable; she never had any previous surgery, and she was not on any medication. The patient’s JOA (Japanese Orthopaedic Association) score was 16 out of 17 (normal function) [6].

**Fig. 1:**
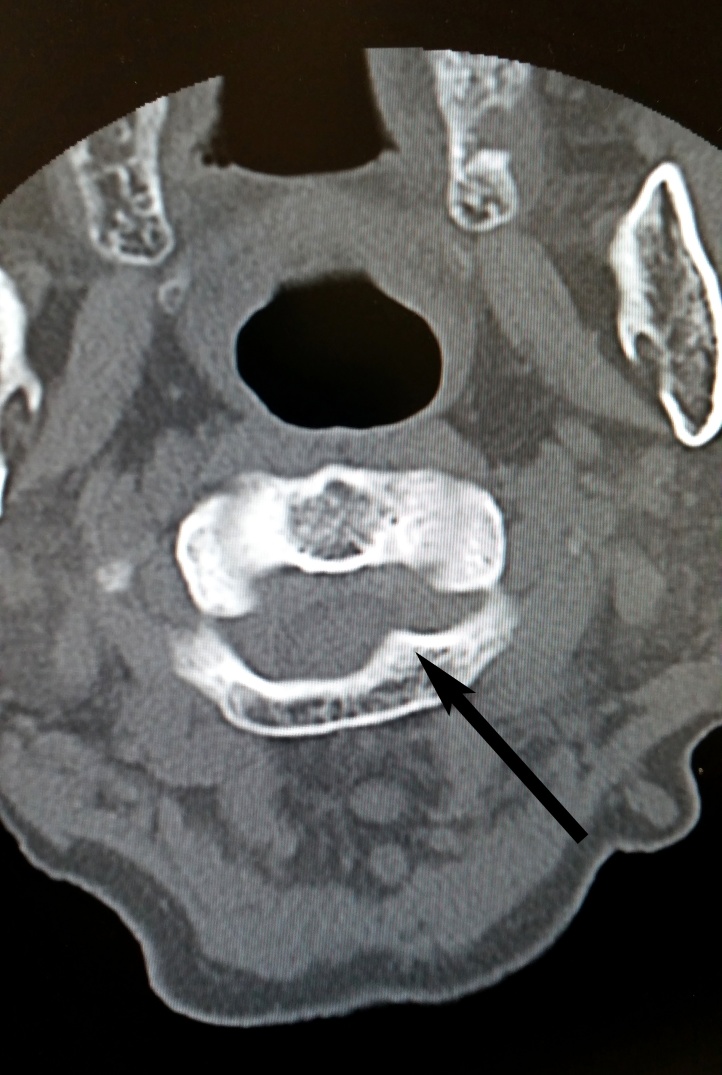
Cervical spine CT-scan showing expansile bone exostosis arising from left posterior arch of C1 vertebra.

**Fig. 2:**
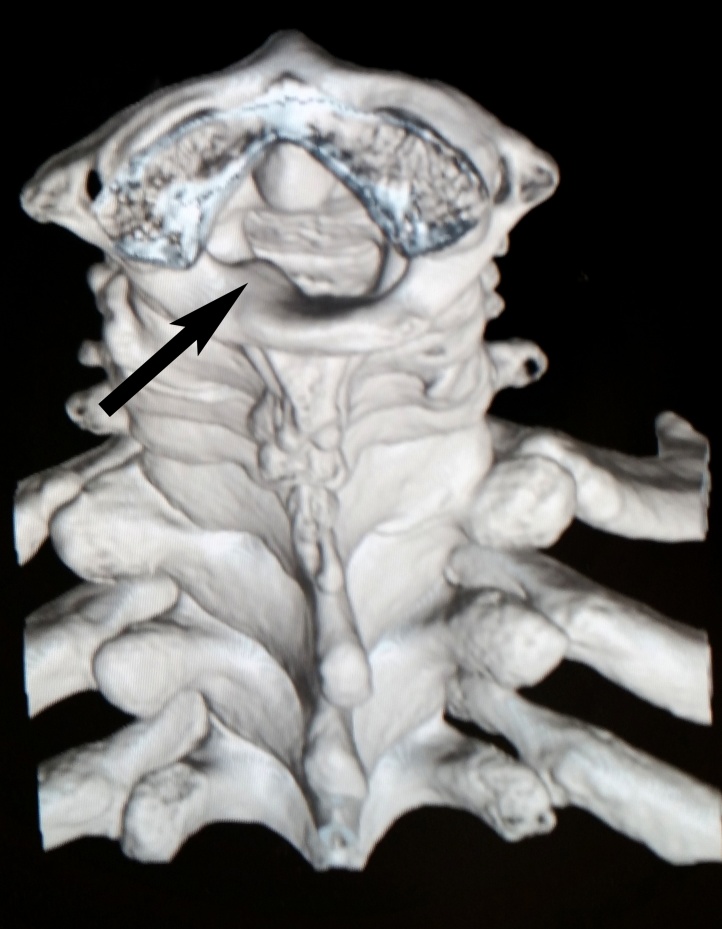
Cervical spine CT-scan showing expansile bone exostosis arising from the left lamina of C2 vertebra.

**Fig. 3:**
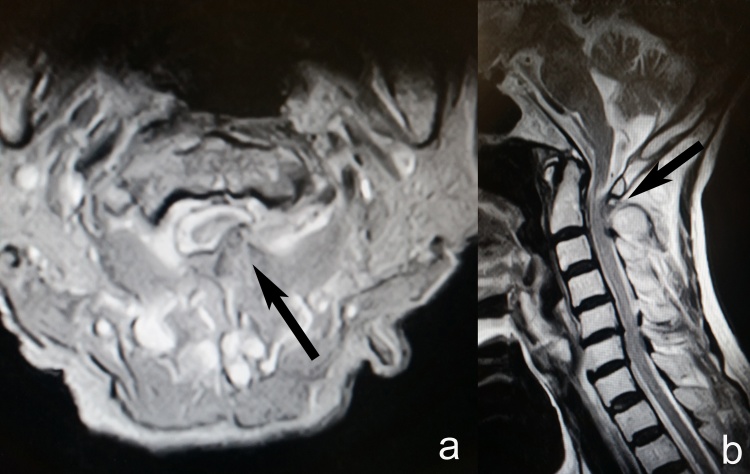
a) Cervical spine MRI showing the lesion (black arrow) on the left side, b) Cervical spine MRI showing the lesion arising from the left lamina of C2 vertebra, no other bony abnormalities noted.

Plain radiographs showed no abnormality. CT-scan and MRI showed expansile bone lesion arising from the left posterior arch of C1 and the left lamina of C2 (Fig. 1, Fig. 2, Fig. 3). It caused posterolateral compression of the spinal cord at the level of the left C1–C2 spinal canal, especially on the left foramina.

Laboratory examinations did not show any abnormality. Based on the clinical features and pathological radiological findings, the authors suggested that the lesion most likely could be an osseocartilaginous benign tumor or known as osteochondroma.

**Fig. 4:**
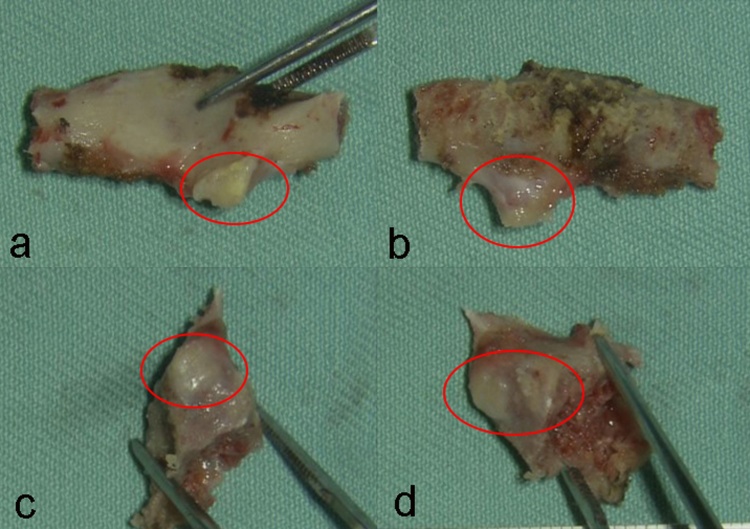
The photographs of the excised lesion taken postoperatively: the posterior arch of C1 verterba, a) ventral, b) dorsal side, and lamina of C2 vertebra, c) lateral, d) medial side. The red circle marks the lesion.

The authors decided to perform decompression through excision of the lesion. The lesion was explored through posterior approach, exposing level of C1 and C2. As C1 lateral mass was preserved, posterior arch osteotomy of C1 was performed. The osteochondral lesion of left lamina C2 was removed by left hemilaminectomy. The lesion excised is depicted in Fig. 4.

Diagnosis of osteochondroma was verified histopathologically. The patient could mobilize on the next day. There was no postoperative wound complication found. The clinical follow-up was performed up to seven days after surgery. The neck pain decreased, and the hypoesthesia of C2–C3 dermatome was significantly improved.

**Fig. 5:**
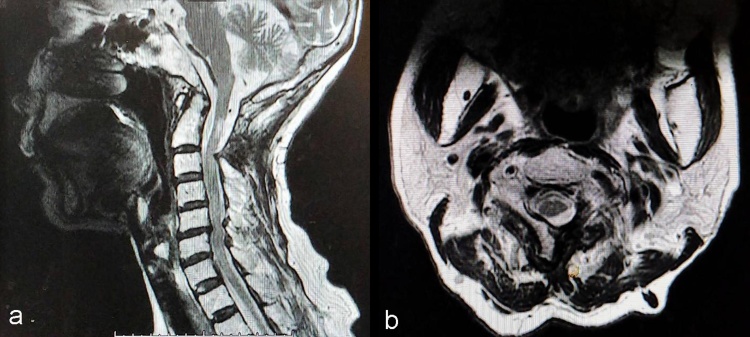
Cervical spine MRI nine months after the operation, there was no sign of recurrence.

Radiologic follow-up by MRI after six months showed no sign of recurrence (Fig. 5). The latest clinical follow-up was 18 months after the operation. The patient was ambulatory, but she complained of mild fingers numbness and moderate neck pain.

## 3 Discussion

Osteochondroma is the most common benign lesion of the bone. It may be multiple or solitary; the former is caused by mutation of exostosin-1 (EXT1) and exostosin-2 (EXT2) [7]. The later can sometimes be caused by a mutation in EXT1 gene, but the exact etiology of this solitary form is still debated [8].

The majority of osteochondroma arises in the metaphyseal region of long bone [9]. It may also arise in the axial skeleton including the spine, in which the most common site affected is on the cervical part. Lotfinia et al. stated that the most common location affected is the C1 vertebrae, contrary to the previous finding which concluded C2 was the most common site [10]. In this patient, there were multiple osteochondromas involving both C1 and C2 vertebrae.

The incidence of this lesion peaks during the third decade of life and usually cease to grow with the closure of growth plate during the adolescent [11]. Thus, this lesion is rarely observed in the elderly such as in this case. Some previous case reports suggest that the development of this lesion may continue beyond skeletal maturity, and it may be affected by other disease progression such as psoriatic arthritis [4].

About 29.5% of all osteochondroma of the spine would cause radiculopathy and about 27% would cause myelopathy. Moreover, the tumor growth may present as a progressive symptom not unlike degenerative changes of spinal stenosis [3]. In the present case, the patient presented with radiculopathy of C1–C2 without any myelopathy.

Other benign tumors of the spine which may occur in older patients includes neurofibroma and giant cell tumor (GCT) [12]. The gradual progression of patients’ symptom may reflect other causes of radiculopathy in the geriatrics, such as ossified posterior longitudinal ligament (OPLL), which is quite common in East Asian population and often affecting the cervical spine [13].

The imaging of spinal osteochondroma usually involves multiple modalities. Conventional plain radiograph is of low diagnostic value, especially for small lesion which is often obscured by adjacent structures [14]. Likewise, in the present case, conventional radiograph didn’t show any abnormality.

Computed tomography (CT) is the gold standard given the lesion’s major osseous component. CT could define its exact location and its connection to the central canal and neural foramen. On MRI, the cortex would be low intensity in any sequence. The marrow would show high intensity in T1 and intermediate intensity in T2. On the other hand, the cartilage cap would show variable intensity according to its degree of calcification [14].

The treatment of this lesion usually involves the *en bloc* resection from the posterior approach (80.5%) due to its location propensity [3]. In this case, the authors also performed an excision through posterior approach. The posterior arch osteotomy and the laminectomy of C2 improved the patient’s symptom of suboccipital pain one day after the procedure.

Since 1987, 13 cases of cervical spine osteochondroma had been reported (Table 1) [4,15,[24], [25], [26],[16], [17], [18], [19], [20], [21], [22], [23]]. The age of presentation ranges from 60 to 77 years old. From those cases, nine were female patients and four were male. This is quite contrary to the findings of various previous studies whereby osteochondroma is more common in males [3,8].

**Table 1:** Literature Review of Cervical Spine Osteochondroma in the Elderly.

Case	Reference	Age/Sex	Chief complaint	Symptom Duration	Neurological Examination	Osteochondroma Location	Surgery	Results
Lozes et al.	1987 [15]	76/F	Right brachial monoparesis	3 months	C5 hypoesthesia Ataxia, spasticity	C4–C4 right foramen	*En bloc* resection + SF	No recurrence after 3 years
Tajima et al.	1989 [16]	62/F	Numbness of upper extremities	3 weeks	Increased triceps, brachioradialis, and legs reflexes; Positive Hoffman sign; Motor weakness up to C6, right side more prominent	C5 posterior arch	HL	Slight hypoesthesia and motor weakness at four years follow-up
Prasad et al.	1992 [17]	60/M	Intermittent neck pain	2 months	Normal	C3 right lamina and pedicle	N/A	No growth in a 6-months follow-up
Ratliff and Voorhies	2000 [18]	66/F	Right lower extremity and truncal numbness	6 months	Hypoesthesia of right T4 and lower dermatome	C5 lamina	L	Sensory improvement but persistent motor weakness after 6-months
Kaneko et al.	2000 [19]	73/F	N/A	N/A	N/A	N/A	N/A	N/A
Sakai et al.	2002 [20]	61/M	Numbness and atrophy of left hand	2 years	Decreased left hand grip strength; Hypoesthesia on bilateral soles	C6 inferior articular facet	L	Improvement of both motor and sensory deficit at 6-months follow-up
		68/F						
				6 months				
			Low back pain and numbness of left hip and thigh					
					Hypoesthesia of the left L4 dermatome			
Akagi et al.	2003 [21]	67/F	Vertigo	10 years	Tenderness on area supplied by greater occipital nerve	C2 lamina	N/A	N/A
Gille et al.	2004 [22]	73/M	Incomplete tetraplegia	N/A	Myelopathy	C2 posterior arch	L	Complete resolution
Yoshida et al.	2006 [23]	61/F	Obstructive sleep apnea	8 years	Normal	C1	A	Complete resolution
Yagi et al.	2009 [4]	77/F	Gait disturbance; neck pain	5 years	Bilateral motor deficit and spasticity. Hyperreflexia of upper and lower limb, left dominant	C1 posterior arch	HL	Complete resolution after 1-month. No recurrence after two years.
Wong et al.	2013 [24]	65/M	Dysphagia	2 months	No abnormality	C2 anterior	A	Complete resolution
Castro-castro et al.	2014 [25]	74/F	Left hemiparesis and torticollis	N/A	Left torticollis; Muscle strength of 4/5 on the left upper and lower extremity	C3–C4 facet joint	HL + SF	Progressive improvement without sequelae
Sciubba et al.	2015 [26]	65/M	N/A	N/A	N/A	C3-T2	*En bloc* resection; approach N/A	No recurrence

A: anterior approach, HL: hemilaminectomy, L: laminectomy, SF: Spinal fusion.

Symptoms duration and characteristics are variable. The shortest presentation was two months, ranging up to ten years. Most cases presented as radiculopathy or myelopathy. There are three unusual presentations of vertigo, obstructive sleep apnea, and dysphagia [21,23,24]. Seven cases were successfully treated without any remaining symptoms or recurrences. Two cases had remaining neurologic symptoms; one case was not treated surgically, but no further growth was observed during follow-up. One case didn’t report the follow-up.

## 4 Conclusion

Osteochondroma of the cervical spine is quite rare, especially in elderly patients. However, this diagnosis could be considered as a cause of progressive radiculopathy in the elderly. Precise diagnosis through careful history taking, physical examination, and multimodal radiologic examinations should be made to solve this problem.

## Declaration of Competing Interest

All authors declare that there is no conflict of interest regarding this study.

## Funding

This study is solely funded by the authors.

## Ethical approval

This study has been reviewed by the authors’ Institutional Review Board, and the patient had given a written consent.

## Consent

The patient had given a written consent. All identifying details have been omiited from the manuscript.

## Registration of research studies

This case report is not registered.

## Guarantor

Andhika Yudistira.

## Provenance and peer review

Not commissioned, externally peer-reviewed.

## CRediT authorship contribution statement

**Andhika Yudistira:** Conceptualization, Data curation, Formal analysis, Funding acquisition, Investigation, Methodology, Project administration, Resources, Supervision, Validation, Writing - original draft, Writing - review & editing. **Yasushi Fujiwara:** Data curation, Formal analysis, Project administration, Resources. **William Putera Sukmajaya:** Conceptualization, Formal analysis, Investigation, Methodology, Software, Writing - original draft, Writing - review & editing. **Ray Asaf Hexa Pandiangan:** Writing - review & editing. **Muhammad Abduh:** Writing - review & editing.
